# *Bacillus* as Premier Biocontrol Agents: Mechanistic Insights, Strategic Application, and Future Regulatory Landscapes in Sustainable Agriculture

**DOI:** 10.3390/plants15030516

**Published:** 2026-02-06

**Authors:** Eduardo Hernández-Amador, David Tomás Montesdeoca-Flores, Juan Cristo Luis-Jorge

**Affiliations:** Department of Botany, Ecology and Plant Physiology, Area of Plant Physiology, Science Faculty, University of La Laguna, Avenida Astrofísica Francisco Sánchez s/n, 38200 San Cristóbal de La Laguna, Santa Cruz de Tenerife, Spain; dmontesd@ull.edu.es

**Keywords:** plant growth-promoting rhizobacteria, *Bacillus*, biocontrol, sustainable agriculture, biostimulant

## Abstract

Agricultural productivity currently faces challenges such as soil fertility issues, climatic instability, pests and diseases, and anthropization. This drives a shift towards sustainable agricultural practices, including biopreparations—products derived from living organisms or their metabolites that serve as biofertilizers, biopesticides, biostimulants, or biodegradation agents. Among these, the genus *Bacillus* is a primary candidate for sustainable agriculture; however, this review primarily covers rhizosphere-isolated organisms referred to as plant growth-promoting rhizobacteria. *Bacillus* strains possess a suite of direct and indirect mechanisms to promote plant development and biocontrol, as well as to tolerate various abiotic stresses. This review aims to describe all the mechanisms attributed to strains of this genus and their impact on different crops to promote plant growth, hormonal regulation (indole-3-acetic acid (IAA), abscisic acid (ABA), and ethylene), tolerance to abiotic stresses such as drought, heavy metals, salinity and heat stress, as well as resistance to pests and diseases. Furthermore, this work analyzes quantitative data regarding yield improvements and the environmental variables that influence the consistency of *Bacillus* performance in the field. Finally, to provide a balanced perspective, the review incorporates future directions in research on biosafety and risk assessment frameworks.

## 1. Introduction

Since the beginning of agriculture, humans have faced numerous challenges, including adverse weather conditions, soil-related issues, and crop diseases. Today, rapid global population growth is the most significant challenge, creating immense pressure to produce enough food while sustainably conserving resources [1]. Building on this recognition, many international organizations are now formulating solutions. In response to these pressures, they have called for a 70–100% increase in agricultural production compared to current levels. Recent estimates predict a 30% population increase by 2030 and a total of 9.7 billion people by 2050 [2]. In line with these international recommendations, the United Nations [3] designated fighting hunger and ensuring food security as a top sustainable development goal. One target is to “ensure the sustainability of food production systems and implement resilient agricultural practices that increase productivity and production, contribute to ecosystem maintenance, strengthen adaptive capacity to climate change, extreme weather, droughts, floods, and other disasters, and progressively improve soil and land quality”. However, the pursuit of increased productivity has paradoxically undermined these sustainability principles. Given production demands and limited cultivable land, intensive agriculture has become the dominant approach to maintaining food supply, emphasizing efficient pest, disease, and crop nutrition management [4]. This tendency has directly contradicted UN sustainability goals through widespread reliance on synthetic fertilizers and pesticides, often deployed without adequate management or regulatory oversight [5,6]. These chemical-intensive practices degrade soil quality—directly opposing the UN target of progressively improving soil and land quality—while introducing contaminants such as heavy metals that threaten ecosystems and human health. Nitrogen fertilizers contribute to soil acidification, salinization, nitrate accumulation, acid rain, and increased plant disease susceptibility [7], while excess nutrient applications cause eutrophication, undermining ecosystem maintenance—another core UN objective. The resulting environmental degradation, coupled with substantial economic and energy costs [8,9], creates a fundamental tension: intensive agriculture temporarily increases production but systematically erodes the ecological foundation required for long-term food security.

Reconciling this contradiction requires a paradigm shift toward agricultural systems that simultaneously achieve productivity and sustainability goals. The FAO defines sustainable agriculture as “the management and conservation of natural resources, oriented toward technological change, ensuring continued satisfaction of needs for present and future generations. It conserves land, water, and genetic resources, does not degrade the environment, and is technically appropriate, economically viable, and socially acceptable” [10]. Operationalizing this vision requires the integration of sustainable practices including crop rotation, cover crops, reduced or no tillage, precision agriculture, integrated nutrient management, and biological pest control [11]. Among these strategies, harnessing native soil microorganisms as biofertilizers, biostimulants, and biocontrol agents represents a particularly promising avenue that directly addresses the intensive agriculture-sustainability paradox, as these organisms mobilize nutrients, produce phytohormones, and enhance stress resistance [12] while maintaining soil health and ecosystem integrity; thus, they fulfill the UN sustainability criteria while meeting productivity demands

This review focuses on one of the most prevalent and widely exploited groups of plant growth-promoting rhizobacteria: the genus *Bacillus*. This work provides a comprehensive overview of *Bacillus* as a plant growth-promoting rhizobacteria (PGPR), fulfilling roles classified as biofertilizers, biostimulants, and biocontrol agents. We analyze both direct and indirect mechanisms that confer plant resistance. The direct mechanisms are discussed first to understand how they facilitate nutrient incorporation and participate in the production of plant phytohormones and siderophores. On the other hand, the indirect mechanisms are presented in terms of their use in tolerating abiotic (saline, thermal, drought, water deficit, and heavy metal accumulation) and biotic stresses. The following section presents the strategies used by *Bacillus* strains to improve crop production. Finally, the future prospects and challenges involved in strain selection, scaling, formulation challenges, biosafety, legislative regulation, and integration into crop systems of Bacillus-based biopreparations are discussed.

## 2. Rhizosphere and Rhizobacteria

The term “rhizosphere” was initially coined by German scientist Lorenz Hiltner [13], defining it as the soil compartment directly influenced by plant roots. This zone’s spatial extent varies depending on factors such as plant species, age, and root morphology, typically ranging from 2 to 80 mm from the root surface [14]. The rhizosphere is described as the storehouse of microorganisms in the soil zone surrounding plant roots, where chemical and biological interactions that play a beneficial role in plant growth take place [15]. The characteristics of this environment are shaped by the soil’s physicochemical properties—including its structure, pH, and nutrient availability—which condition microbial proliferation. The rhizosphere is divided into three zones: the endorhizosphere, which consists of the root cortex and endodermis where the microorganism and nutrients reside between plant cells; the rhizoplane which is the middle zone consisting of epidermal root cells, cortex and mucilage cells; and the ectorhizosphere which is the soil immediately adjacent to the root [16].

Crucially, the rhizosphere is profoundly influenced by substances secreted by the plant roots, known as root exudates. These exudates—which include sugars, organic acids, phenolic compounds, enzymes, phytohormones, and vitamins—act as signaling molecules that can either stimulate or inhibit the growth of organisms in this environment [17]. Hence, it harbors an extremely complex microbial community, and it includes saprophytes, endophytes, epiphytes, and pathogens as well as many useful microorganisms like bacteria, fungi, nematodes, protozoa, algae, etc. [18]. Root exudates, low-molecular-weight compounds such as organic acids, vitamins, and amino acids, specifically select for microorganisms capable of metabolizing them, thereby shaping the diverse and specialized bacterial community found here. Interactions within this zone can be beneficial, such as growth stimulation, or harmful, such as infection by pathogens or viruses [19]. This specialized bacterial community is often referred to as rhizobacteria. Those that promote plant growth are known as plant growth-promoting rhizobacteria (PGPR). The concept of PGPR originated with the discovery of bacteria capable of combating plant pathogens [20]. Today, the term encompasses bacteria that benefit plant development through various mechanisms.

PGPRs are commonly categorized based on their primary functions. Biofertilizers are PGPR that enhance nutrient availability, typically through nitrogen fixation or the solubilization of key nutrients, or by producing siderophores [21]. Biostimulants activate physiological and molecular processes, thus modulating plant performance and quality, often through the biosynthesis of phytohormones like auxins, cytokinins, gibberellins, abscisic acid, and ethylene [22,23]. Biocontrol Agents are natural or modified microorganisms defined as reducing the incidence or severity of diseases caused by plant pathogens, often replacing chemical pesticides [24]. It should be noted that many PGPR possess characteristics that allow them to be classified simultaneously across these categories (biofertilizers, biostimulants, and control agents). However, some regulatory bodies, such as the European Union (EU), employ a more restrictive definition. Within the context of EU regulation (EU; 2019/1009) [25], a ‘plant biostimulant’ is defined as an EU fertilizing product whose function is to stimulate plant nutrition processes independently of its nutrient content, with the sole aim of improving specific characteristics, including: nutrient use efficiency, tolerance to abiotic stress, quality traits, or the availability of confined nutrients in the soil and rhizosphere. Therefore, within EU borders, the term ‘biostimulant’ must strictly comply with these defined characteristics.

An essential aspect of PGPR functionality concerns the spatial localization of bacterial populations relative to plant tissues—whether bacteria function as epiphytic colonizers of root surfaces (rhizoplane), inhabitants of the surrounding rhizosphere soil, or as endophytic colonizers of internal plant tissues. Many Bacillus species exhibit facultative endophytic capacity, colonizing not only the rhizosphere and rhizoplane but also penetrating root tissues to establish populations within intercellular spaces, vascular tissues, and occasionally aerial plant parts [26,27].

Among PGPR, the genus *Bacillus* has emerged as one of the most extensively studied and commercially exploited groups. *Bacillus* spp. and *Pseudomonas* spp. represent the most predominant rhizobacteria in agricultural soils [15], with *Bacillus* accounting for up to 95% of the Gram-positive bacterial population in these habitats [28]. The ecological success of *Bacillus* in the rhizosphere is due to several features that will be discussed in the following section. These characteristics, combined with their multifunctional PGPR traits have positioned *Bacillus* species as key components in sustainable agricultural practices. This convergence of ecological fitness and plant-beneficial properties makes *Bacillus* an ideal model for understanding PGPR-plant interactions and developing next-generation bioinoculants.

## 3. Genus Bacillus

This genus comprises more than 300 species and subspecies within the phylum *Firmicutes* [29,30]. These bacteria possess a bacillary morphology, exhibit flagellar motility, and are variable in size [31]. Regarding growth conditions, they are mostly mesophilic, with optimal growth at neutral pH. They can grow aerobically and often function as facultative anaerobes [32]. The remarkable proliferation and presence of *Bacillus* in diverse habitats are secured by two key attributes: their metabolic and genetic diversity, and their ability to develop endospores resistant to adverse environmental conditions (including the absence of water and nutrients, temperature extremes, UV radiation, and unfavorable pH) [5,33,34]. Endospore resistance makes them ideal for biopreparation formulations because their storage and viability are not compromised, giving them a competitive advantage over other microorganisms [5]. Furthermore, *Bacillus* species exhibit biofertilizing and biostimulating properties, but this genus is the most widely exploited for pest and disease control due to its biocontrol activities [29,33,35]. These bacteria are known for producing a variety of metabolites (lytic enzymes, volatile organic compounds, siderophores, and toxins) that can inhibit the growth and cellular functions of target organisms, including bacteria, fungi, nematodes, insects, and even viruses [36]. Examples of industrially relevant species used in commercial preparations include *B. subtilis*, *B. amyloliquefaciens*, *B. pumilus*, *B. licheniformis*, *B. megaterium*, *B. velezensis*, *B. cereus*, *B. thuringiensis*, *B. firmus*, and *B. mycoides* [28,37,38,39,40]. Specifically, in Spain, numerous phytosanitary products based on 20 strains of five *Bacillus* species are distributed—namely, *B. firmus*, *B. subtilis*, *B. amyloliquefaciens*, *B. pumilus*, and *B. thuringiensis* [41] (Table 1).

## 4. Plant Growth Promotion Mechanisms of Bacillus Genus

**Figure 1:**
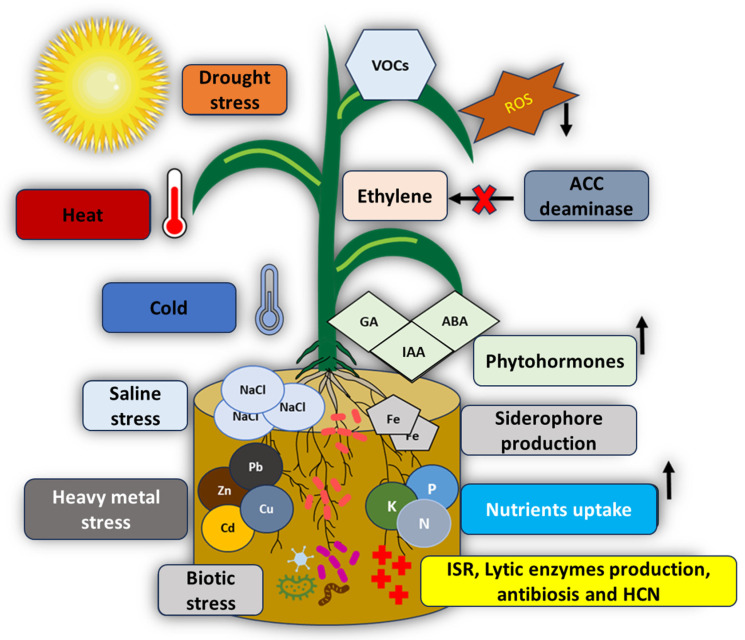
On the **left** are some of the biotic and abiotic stresses that affect plant development. On the **right** are the direct and indirect mechanisms that promote plant growth, abiotic stress tolerance and biocontrol: phytohormones, P solubilization, K solubilization and nitrogen fixation, siderophore production, lytic enzyme production, induced resistance system, biocontrol of pathogens and diseases, and control of abiotic stresses. Exists an improvement in nutrition uptake and phytohormone production. The arrows shows that increase. On the other hand, ROS are decreased.

The mechanisms employed by plant growth-promoting rhizobacteria (PGPR) are classified as direct or indirect, as presented in Figure 1. However, beyond this categorical distinction, these mechanisms function as interconnected physiological processes that collectively establish a dynamic plant–microbe partnership responsive to environmental conditions.

Direct mechanisms constitute biological interventions that enhance nutrient acquisition, mobilization, and bioavailability for the plant. These include nitrogen fixation (conversion of atmospheric nitrogen into plant-available forms), solubilization of phosphorus and potassium—macronutrients essential for plant development [34,42,43,44]—and the synthesis of phytohormones such as auxins, cytokinins, and gibberellins, which modulate plant developmental programs [28,29,30,35]. Additionally, PGPR produce siderophores, high-affinity iron-chelating compounds that enhance micronutrient availability under iron-limiting conditions [44,45]. Critically, these direct mechanisms do not operate in isolation but form a coordinated nutritional and hormonal provisioning system that adapts to soil biogeochemical properties and plant physiological status.

On the other hand, indirect mechanisms enhance plant resilience to biotic and abiotic stressors through a hierarchical defensive repertoire. Under stress conditions—whether abiotic (drought, salinity, etc.) or biotic (pathogen attack)—PGPR deploy protective responses including antibiotic and antifungal compound production, synthesis of lytic enzymes, accumulation of osmolytes for cellular osmoprotection, emission of organic volatile compounds with antimicrobial properties, biofilm formation as physical barriers, and elicitation of plant-intrinsic defense systems [46,47,48]. The deployment of these mechanisms is context-dependent and calibrated to stressor type and intensity, reflecting the sophisticated nature of plant-PGPR communication mediated by root exudates and bacterial signaling molecules.

This integrated perspective—wherein direct and indirect mechanisms function as complementary modules within a bidirectional plant-bacteria dialog—provides the conceptual foundation for understanding how specific *Bacillus* strains confer multifaceted agronomic benefits under diverse field conditions.

### 4.1. Direct Mechanisms

#### 4.1.1. Nutrition Uptake

Nitrogen fertilizers are frequently used to supplement this vital nutrient for plants. However, improper application leads to contamination problems. Plant growth-promoting rhizobacteria (PGPR) offer an alternative to reduce chemical product use. Some PGPR provide nitrogen to plants by biologically fixing atmospheric nitrogen into ammonium, using the nitrogenase enzyme. This process makes nitrogen available to the roots. The nifH gene is involved in atmospheric nitrogen fixation, and has been found in strains such as *B. aerophilus*, *B. oceanisediminis*, *B. safensis*, and *B. flexis* [49]. Biological nitrogen fixation can occur both symbiotically and non-symbiotically [50]. Nitrogen-fixing strains reported in the literature include *B. cereus*, *B. circulans*, *B. firmus*, *B. pumilus*, *B. licheniformis*, *B. megaterium*, *B. subterraneous*, *B. aquimaris*, *B. vietnamensis*, *B. aerophilus*, and *B. siamensis* [26,35,51].

In vivo studies on corn (*Zea mays*) treated with *B. pumilus* showed improved nitrogen content and increased dry biomass [52]. In Japan, researchers isolated *B. altitudinis* from the rhizosphere of rice (*Oryza sativa*). This strain exhibited nitrogen-fixing activity and served as a basis for the development of native rice biofertilizers [53]. The application of *Bacillus* rhizobacteria shows promising results, which could reduce nitrogen fertilizer use.

Many *Bacillus* rhizobacteria can solubilize phosphorus, transforming insoluble forms into soluble ones. They possess the phosphatase enzyme and release organic acids that make phosphorus in the soil more available to plants [54]. In recent studies, *B. safensis* has been shown to improve wheat (*Triticum aestivum*) yield and growth in both greenhouse and field conditions by solubilizing phosphorus and producing indole acetic acid [55]. In China, phosphorus-solubilizing *Bacillus* sp. increased the size and biomass of striped-stalked saltwort (*Suaeda salsa*) [56].

The DYS211 strain was also tested. In garlic (*Allium sativum*), a mix of *Pseudomonas* sp. and *Bacillus* sp. increased leaf phosphorus and boosted plant growth, resulting in greater size and dry biomass than controls without phosphate fertilizer [57]. In greenhouse-grown corn, the P-solubilizing *B. altitudinis* strain improved phosphorus uptake. This led to higher seed germination, shoot size (155%), root size (45%), and dry biomass compared to plants without bacterial inoculation [58]. Other *Bacillus* species such as *B. circulans*, *B. cereus*, *B. fusiformis*, *B. pumilus*, *B. megaterium*, *B. mycoides*, *B. coagulans*, *B. chitinolyticus*, *B. subtilis*, and *B. siamensis* have also been identified as phosphorus solubilizers [26,35,51].

Potassium, along with phosphorus and nitrogen, is an essential macronutrient for plants. It plays roles in photosynthesis, stomatal regulation, seed development, and crop growth [29,59]. While potassium is abundant in soil, only 1% to 2% is available to plants [60]. Over 90% exists as silicate minerals and insoluble rocks [48,61]. Finding local soil sources for potassium enrichment is difficult. PGPR is a promising tool that can solubilize potassium by releasing organic acids. These acids convert insoluble potassium from minerals into forms plants can absorb [29,62]. Many *Bacillus* strains exhibit potassium-solubilizing activity. Examples include *B. velezensis*, *B. cereus*, *B. circulans*, *B. coagulans*, *B. edaphicus*, *B. megaterium*, *B. subtilis*, *B. firmus*, *B. mycoides*, *B. decolorationis*, *B. horikoshi*, and *B. siamensis* [26,51,63]. Using these bacteria improves plant development. For instance, *B. cereus* increased dry biomass and height in tomato (*Solanum lycopersicum*) [64]. *Bacillus*-mediated potassium solubilization has also boosted theanine production in tea (*Camellia sinensis*) by activating the responsible synthesizing enzyme [65]. In one experiment, *Bacillus* spp. mobilized 53.6% to 304.8% of soil potassium into wheat seeds under greenhouse conditions [66]. *Bacillus* sp. INCA-FRc7 and INCA-FRc19x strains from corn rhizosphere solubilized potassium from feldspar and muscovite under varying conditions [67]. *B. licheniformis* and *B. cenocepacia* also showed robust potassium-solubilizing activity and improved tomato growth, increasing size, weight, leaf area, and biomass [68]. These findings support the use of *Bacillus* rhizobacteria in potassium solubilization, promoting more sustainable and environmentally friendly agriculture.

The analysis of nutrient acquisition mechanisms reveals a hierarchical pattern in both research intensity and agronomic applicability among *Bacillus*-mediated strategies. Phosphorus solubilization emerges as the most extensively documented function with species exhibiting this trait (*B. safensis*, *B. circulans*, *B. cereus*, *B. megaterium*, *B. subtilis*, *B. siamensis*), reflecting the global prevalence of P-limiting soils and the uses of phosphatase production and organic acid secretion across the genus. In contrast, biological nitrogen fixation, despite its high agronomic value, appears restricted to fewer species (*B. pumilus*, *B. cereus*, *B. aerophilus*, *B. siamensis*), likely due to the substantial energetic costs associated with nitrogenase activity and the complex regulatory networks governing *nif* gene expression. Potassium solubilization occupies an intermediate position, with documented activity in species such as *B. velezensis*, *B. cereus*, *B. megaterium*, and *B. subtilis*, although field-scale validation remains limited compared to P-solubilization studies [26,35,51,62].

Critically, emerging evidence points toward multifunctional strains capable of simultaneous mobilization of multiple macronutrients—*B. megaterium* and *B. cereus* exemplify this synergistic capacity, combining P- and K-solubilization with varying degrees of N-fixation potential. Such multifunctional profiles generate additive or synergistic effects on plant biomass accumulation, particularly in polydeficient soils where single-nutrient amendments prove insufficient. However, the translation of these mechanisms from controlled conditions to heterogeneous field environments requires addressing critical knowledge gaps: (i) competitive fitness of inoculated strains against indigenous microbiota with similar functions, (ii) persistence and activity maintenance across varying soil physicochemical conditions, and (iii) quantification of actual nutrient flux from bacterial solubilization to plant uptake under realistic agricultural scenarios. The predominance of greenhouse studies over multi-location field trials underscores the need for agronomic validation that integrates soil type, indigenous microbial communities, and crop-specific responses into predictive frameworks for inoculant performance.

#### 4.1.2. Phytohormone Production

Numerous studies confirm the biostimulant capacity of *Bacillus* genus strains through the production or modulation of key growth regulators/phytohormones, including auxins, cytokinins, gibberellins, abscisic acid, and ethylene. The synthesis of these compounds is closely related to their involvement in physiological processes that support overall plant growth and development [23,29,30,40,41,69].

Within the auxin group, indole-3-acetic acid (IAA) is the predominant phytohormone. This compound plays a key role in several developmental processes, promoting apical dominance, root elongation, and cell division. IAA is also deeply involved in flowering, seed germination, senescence, and defense systems [23]. The biosynthesis of IAA often utilizes L-tryptophan as a precursor, which the plant secretes, and the rhizobacteria then use [69,70]. The capacity of the *Bacillus* genus to produce auxins significantly enhances plant development. Specific examples detailing IAA production levels, strain efficacy, and target crop responses, including under high, controlled, or stress conditions, are compiled in Table 2. Moving on from auxins, cytokinins represent another critical class of hormones that participate in essential physiological processes. These compounds primarily promote cell division, regulate stomatal opening, aid in chloroplast differentiation, and delay leaf senescence. Cytokinins are also involved in vascular development, germination, and plant-pathogen interactions [40,71,72]. Several strains of *Bacillus* species have been shown to produce cytokinins, such as zeatin and isopentenyladenine. These positive effects on cytokinin biosynthesis led to increased cotyledon size and weight, as well as improved lateral root growth, mainly by inducing cell division, thereby enhancing plant development across several species (Table 2).

On the other hand, gibberellins (GAs) are essential phytohormones that play a principal role in plant development and physiological processes. Their activity focuses on shoot and root development, seed germination, flowering, and fruit development [45,97,98]. Low levels of GAs can result in smaller plants compared to those with normal levels. Among the most critical forms are GA3 (gibberellic acid) and GA1, which are highly active in plants [69]. Gibberellin production by *Bacillus* strains is also associated with increased nutritional content in plant tissues, including various amino acids, macro- and micronutrients, organic acids, fructose, and carotenoids [99]. Strain-specific evidence linking *Bacillus* gibberellins production has shown positive effects on plant development and resistance to environmental stresses in several crop species (Table 2) [23]. Turning next to abscisic acid (ABA), this hormone plays a crucial role in regulating different stages of germination, fruit abscission, and stomatal opening [29,100]. Additionally, ABA participates in senescence processes and, critically, regulates responses to abiotic stresses, notably salinity, thermal stress, and heavy metal exposure [12,29,30]. Several *Bacillus subtilis* strains have been identified as producers or regulators of this phytohormone with increased tolerance to stresses such as salt and heavy metals [89,92]. In addition, indirect effects have been reported, particularly in IAA production and changes in macro- and micronutrient content, demonstrating its biostimulant potential [90].

Finally, ethylene, a phytohormone that regulates ripening and senescence in climacteric fruits [29,30,69], also controls numerous stress responses—including those triggered by a saline, thermal, or heavy metal presence—and regulates plant development processes like seed germination and organ senescence [12,29,30,69,82,97,101,102]. Instead of directly producing high levels of this hormone, certain *Bacillus* strains help modulate stress-induced ethylene levels [69,97]. They achieve this by synthesizing the enzyme ACC deaminase, which degrades the ethylene precursor ACC, thereby lowering ethylene levels in the plant system [93,94,95,96]. This action enhances plant tolerance, particularly to salt and heat stress (Table 2).

The synthesis of phytohormonal data reveals IAA as the predominant and most ubiquitously produced phytohormone across *Bacillus* species, with documented production in *B. licheniformis*, *B. subtilis*, *B. megaterium*, *B. velezensis*, *B. amyloliquefaciens*, *B. cereus*, *B. thuringiensis*, *B. mycoides*, and *B. siamensis* [73,74,75,76,77,78,79,80,81]. This prevalence reflects the central role of auxins in fundamental developmental processes that directly translate to enhanced nutrient acquisition and biomass accumulation across diverse crop species. The tryptophan-dependent biosynthetic pathway, relying on plant-secreted precursors, positions IAA production as a co-evolved mutualistic trait optimized through rhizosphere dialog. In contrast, cytokinin production appears more restricted taxonomically (*B. subtilis*, *B. toyonensis*, *B. licheniformis*, *B. amyloliquefaciens*), yet exerts profound morphogenetic effects including delayed senescence, enhanced chloroplast development, and lateral root proliferation that complement auxin mediated responses.

Gibberellin biosynthesis, documented in *B. tequilensis*, *B. methylotrophicus*, and select *B. amyloliquefaciens* strains, demonstrates particular relevance under abiotic stress conditions, where GA-mediated activation of stress-protective pathways synergizes with direct growth promotion. Notably, ABA modulation—whether through direct biosynthesis (*B. amyloliquefaciens*, *B. subtilis*, *B. mirasflavi*) or regulatory influence on endogenous plant levels—emerges as a critical mechanism linking biostimulation with stress tolerance, particularly under salinity, drought, and heavy metal exposure. The ACC deaminase pathway represents a distinct strategy wherein *Bacillus* strains (*B. mojavensis*, *B. subtilis*, *B. safensis*, *B. aryabhattai*) mitigate stress induced ethylene accumulation rather than directly synthesizing hormones, highlighting the diversity of hormonal modulation mechanisms.

A critical observation emerges from cross-referencing Table 2: strains exhibiting multi-hormonal production profiles (*B. amyloliquefaciens* producing IAA, cytokinins, GAs, and ABA; *B. subtilis* synthesizing IAA, cytokinins, and expressing ACC deaminase) consistently demonstrate superior field performance compared to single-hormone producers. This suggests that hormonal synergy—coordinated auxin-cytokinin ratios driving balanced shoot–root development and GA-ABA interplay modulating stress responses—constitutes a more effective biostimulation strategy than isolated hormonal effects. However, the mechanistic basis for strain-to-strain variation in hormonal profiles remains poorly understood. Whether these differences reflect genomic variation in biosynthetic gene clusters, differential gene expression in response to specific root exudate profiles, or metabolic trade-offs between hormone production and other PGPR functions requires elucidation through comparative genomics and transcriptomics.

#### 4.1.3. Siderophore Production

Iron is an essential micronutrient and a major limiting factor in plant metabolism and development; its deficiency often leads to reduced crop yields and disease emergence [103,104]. This element is critical for numerous physiological processes, including cellular respiration, electron transfer, chlorophyll synthesis, and DNA and RNA synthesis [28,29,103]. Although iron is widely distributed in the lithosphere, its predominant form—Fe^3+^ (often as silicates, hydroxides, or oxides)—is poorly soluble at neutral or alkaline pH, which severely limits its availability to plants [69]. To address this issue, some plants and microorganisms produce siderophores, which are low-molecular-weight molecules (200–2000 daltons) that chelate metals with high affinity, especially iron. These siderophores sequester insoluble Fe^3+^ and reduce it to soluble Fe^2+^, enhancing its availability for plant uptake. Siderophores are structurally classified into three primary families based on their ligands and functional groups: catecholates, hydroxamates, and hydroxycarboxylates [12,28,105]. Many hydroxamate-type and catecholate-type siderophores, along with those containing nitrogenous heterocycles, are generated from amino acid or aromatic acid units through non-ribosomal peptide synthetases (NRPS). For instance, bacillibactin, a catecholate-type siderophore common in Bacillus, is synthesized via the bacACEBF operon [106]. In contrast, siderophores containing-hydroxycarboxylates are often produced through NRPS-independent pathways, typically involving the adenylation of a carboxylic acid substrate [107].

The function of siderophores extends significantly beyond iron acquisition. They play a pivotal role in biocontrol by reducing iron availability in the rhizosphere, thereby restricting pathogen development [5,29,108]. Furthermore, siderophores produced by plant growth-promoting rhizobacteria (PGPR) can chelate heavy metals such as Cd, Pb, Ni, As, Al, and Cu. This mechanism helps organisms manage heavy metal stress and positions these compounds as potential tools for bioremediation in polluted environments. This metabolism is also closely tied to direct biocontrol, as the NRPS enzymes involved in siderophore synthesis may also generate molecules with antibiotic properties [35,109]. The genus Bacillus is highly notable for producing siderophores. The effectiveness of these compounds in promoting plant growth and stress tolerance has been demonstrated across numerous field and pot trials. For instance, specific data detailing the efficacy under saline stress, increased development, and enhanced biomass and root length in several crops are summarized in Table 3.

### 4.2. Indirect Mechanisms

#### 4.2.1. Control of Abiotic Stresses

Agricultural production is influenced by biotic and abiotic factors, the latter referring to those adverse environmental conditions that negatively impact plant growth and development [112]. Abiotic stresses include drought, salinity, waterlogging, temperature fluctuations and the presence of heavy metals, all of which are capable of altering plant metabolism and physiology [32]. At the present time, when climate change is presented as a threat to agricultural production, the effects of abiotic stresses have become increasingly accentuated according to data collected in recent years. Considering this situation, PGPRs emerge as a viable strategy to alleviate and safeguard plants amidst the escalating prevalence of stressful conditions. This approach aims to uphold sustainable practices and enhance food security amidst growing environmental challenges [30]. The main abiotic stresses and the use of species of the genus *Bacillus* for their tolerance at the plant level will be briefly discussed below. See the Supplementary Materials (Table S1) to find more examples of molecular mechanisms of *Bacillus* PGPR in Plant Abiotic Stress Responses [113,114,115,116,117,118].

##### Salt Stress

Salt stress is a limiting factor in agricultural production, causing nutritional deficiencies, osmotic stress resulting in water deficit, and oxidative stress with the overproduction of reactive oxygen species [119,120]. The plant is consequently affected in most of its physiological processes such as germination, reproduction, and vegetative growth [121].

Soil is considered saline when its saturation extract electrical conductivity is 400 mS m^−1^ (approximately 40 mM NaCl) at 25 °C, and its exchangeable sodium is 15% [122]. The accumulation of high concentrations of soluble salts, such as sulfates and chlorides, in the roots or adjacent areas can be toxic to the plant, and in these situations, it is considered a case of salt stress [123]. In the coming years, soil salinization will take place at an accelerated rate, leading to more arable land becoming saline and hindering the viability of farms, as it is estimated that almost 20% of cultivated land will be affected by salinization [124]. This salinization may depend on natural processes, known as primary salinization, or on anthropogenic processes known as secondary salinization. In reference to the first term, this groups together the physicochemical properties of the soil itself, low precipitation, and high temperatures, which have been increasingly accentuated by climatic change. On the other hand, secondary salinization is due to human practices such as irrigation with water of poor quality, the use of inadequate agricultural techniques and the use of synthetic fertilizers and pesticides, causing a loss of soil fertility and a reduction in agricultural production [125]. Currently, the search for new methods to identify environmentally suitable solutions to maintain production under salt stress is of particular interest. The traditional approach has been based on two avenues, agricultural management practices and plant breeding [122]. Although sustainable soil management can reduce the effects of salinity on crops and the environment itself, since soil is a slow-generating resource, this action is limited by the cost, availability, and quality of water resources. On the other hand, the development of plant breeding is also somewhat costly and slow, since it is not easy to implement [123]. In recent years, the application of *Bacillus* as PGPR has been shown to be effective in improving plant development under adverse situations such as salt stress [125]. PGPRs can alleviate salt stress in plants through several direct and indirect mechanisms such as osmotic regulation, phytohormone production, increased nutrient uptake, ion homeostasis, reduced oxidative stress and enhanced antioxidant activity or secretion of organic volatile compounds [126,127,128].

As a result of focusing on PGPR as an environmentally friendly and economical alternative to combat salt stress, some studies have used strains of *Bacillus* spp. as the main protagonists in the trials (Table 4). Although *Bacillus*-mediated salinity stress amelioration under controlled conditions is compelling, the translation of these laboratory and greenhouse results to heterogeneous field environments reveals critical limitations that constrain commercial deployment. Salt stress in agricultural systems rarely manifests as uniform NaCl concentrations applied at defined developmental stages but rather as spatially heterogeneous soil salinity gradients, temporally variable salt accumulation patterns driven by irrigation practices and evapotranspiration dynamics, and complex multi-ion compositions (Na^+^, Cl^−^, SO_4_^2−^, Mg^2+^, Ca^2+^) that differentially affect both plant physiology and bacterial survival.

##### Temperature Stress

More extreme temperature fluctuations are becoming increasingly common due to climate change sweeping the globe. The effects of this on global agriculture pose a critical threat to production development as plants are affected in their biochemical activities, physiological and molecular processes, and morphology [135]. Plants are sensitive to stress due to high temperatures and cold. With regard to heat, which is accentuated worldwide as a result of global warming, the water potential and relative water content of plants decrease substantially when exposed to this stress [30]. Thus, germination, seed development, cell turgor and other aspects of the plant are affected [136]. On the other hand, consequent cold stress is one of the major environmental factors that restrict the development of any type of plant, posing a risk in maintaining food security [34]. Cold-generated heat stress can limit crop performance by inducing metabolic and physiological disparities leading to ROS accumulation, nutritional disorders, cell membrane function problems, reductions in photosynthetic activity and hormonal imbalances [68,135]. To cope with these changes, plants activate several molecular and physiological processes [137].

Although plants possess their own mechanisms to cope with stress generated by high and low temperatures, the use of PGPR as a protective agent against these thermal changes is increasingly in demand due to its benefits. Rhizobacteria of the genus *Bacillus* are microorganisms that are the protagonists of many studies related to heat-induced stresses since their ability to form endospores at high temperatures makes them bacteria with good thermotolerance. At the other extreme, there are also studies on *Bacillus* species with the ability to improve cold stress tolerance in different cultivars (Table 5).

A critical unresolved question in *Bacillus*-mediated thermotolerance concerns whether temperature stress mitigation capacity represents a conserved species-level trait or reflects strain-specific adaptation requiring individualized screening and validation. The current literature presents conflicting evidence: some studies report thermotolerance as a general characteristic of specific *Bacillus* species (*B. subtilis*, *B. licheniformis*, *B. amyloliquefaciens*), while others document significant intraspecific variation where only particular strains within a species demonstrate functional thermotolerance-enhancing capacity. The current practice of extrapolating efficacy from one strain to all members of a species risks deployment of ineffective inoculants and may explain inconsistent field performance reported in commercial applications.

##### Drought Stress and Water Deficit Stress

Stress generated by drought or water deficit is one of the major determinants of crop yield and productivity [54]. According to estimates for 2030, food production will decrease in many regions of the planet due to the low availability of water and the lack of responsible practices that manage current resources correctly [144]. In addition, the climate change we are experiencing further accentuates this problem by producing extreme temperatures, changes in precipitation patterns, soil salinization and increasingly rapid evaporation that affects all areas of the planet, being particularly harder in arid and semi-arid regions [135,145]. Drought is defined as an abiotic condition characterized by a deficit in the supply of water to the plant with respect to its demand. This means that the transpiration ratio exceeds the water acquisition ratio. Thus, plant development is hindered by a drop in water potential and tissue turgor [136]. The effects of osmotic stress on the plant cause variations in its physiology and metabolism, depending on its severity and duration, as well as on the developmental stage [146]. Likewise, there is a reduction in germination rate, leaf area, cell division and photosynthetic activity, affecting plant growth [144]. On the other hand, water deficiency reduces accessibility to carbon dioxide, generating reactive oxygen species such as superoxide ion, hydrogen peroxide and hydroxyl radicals within plant cells, which in turn can trigger cell apoptosis in severe cases [135,145,147]. In order to cope with the negative impacts, plants have a number of physiological, morphological, cellular, biochemical and molecular processes that allow them to have an adaptive response to drought [148]. However, the use of PGPRs as a sustainable alternative has gained momentum in recent years. Rhizobacteria possess different mechanisms to alleviate osmotic stress and promote plant growth, which have been previously described, as well as the production of osmolytes, antioxidants, changes in root architecture, production of exopolysaccharides, volatile organic compounds and ACC [148,149]. There is now evidence of positive effects on osmotic stress conditions following the inoculation of *Bacillus* PGPR species (Table 6).

##### Heavy Metals Stress

Nowadays, soils are contaminated with heavy metals because of numerous anthropogenic activities such as modern agricultural techniques, which insist on using agrochemicals in an uncontrolled manner to fertilize as well as to combat pests and diseases [137,154]. Moreover, inadequate management of wastewater from industries and its use in the crop irrigation system only elevates the negative effects brought by these pollutants on ecosystems and all their life forms, including humans [155,156]. To understand this issue, first of all, it is necessary to know why an accumulation of heavy metals in soil is dangerous. Heavy metals are high-density elements that are toxic at low concentrations, so their bioaccumulation and low biodegradability make them a worrying threat in the agricultural sector [30,127,157,158,159]. Among the most common due to their toxicity to soil and crops are Cd, Cu, Zn, Ni, Co, Cr, Pb and As [30,160,161]. In the presence of these in limiting concentrations for the plant, damage occurs in the photosynthetic apparatus, cell organelles and cell wall, thus affecting physiology and metabolic activities [68]. The electron transport chain is also affected and ROS are generated which induce oxidative stress on the plant [137,162]. Despite the existence of several adaptive mechanisms in plants to tolerate heavy metals (protein repair, metal chelation and production of antioxidant enzymes), the use of PGPRs is a sustainable and cost-effective reinforcement in stress control [34,158]. PGPRs have the ability to remove toxic metals through mechanisms such as acidification, solubilization and the production of chelating agents called siderophores that sequester and form complexes with these pollutants [147]. The production of phytohormones, exopolysaccharides and antioxidant enzymes also play a key role in tolerance to this stress and in enhancing plant development [127,163]. There is evidence of how beneficial the application of rhizobacteria is in crops affected by the presence of heavy metals, either in vivo or in vitro (Table 7).

#### 4.2.2. Biotic Control

Biotic stress conditions affect crop growth, development and yield, with estimated losses of 30% in agricultural productivity [168]. To deal with pathogens and diseases that interfere negatively with plant health, chemicals known as pesticides are used. However, their mismanagement throughout the history of their application has generated resistance to them, as well as environmental contamination and high economic costs that have repercussions on crops, soils, the ecosystem, consumers and the producer’s own pocket [169]. Faced with this problem, the biological approach, i.e., the use of living organisms to treat plant pathogens and plant diseases is presented as a necessary and sustainable measure [15]. Thus, the term biocontrol arises, where the capabilities of living organisms allow the development of pests or diseases generated by pathogenic species to be reduced [170]. PGPR have gained relevance in this regard, as they present several tools that allow them to manage the control of pathogens and ensure optimal conditions for plant development [48]. These tools include lytic enzyme production, induced resistance system activation, antibiotic production, biofilm formation, quorum sensing, volatile organic compound production, and hydrogen cyanide production among others [149,170,171,172] (Table 8).

##### Lytic Enzymes

The production of lytic enzymes by PGPR belonging to the genus *Bacillus* is one of the defense and prevention mechanisms against phytopathogens, especially those of a fungal nature [46]. Among the lytic enzymes produced amylases, cellulases, chitinases, glucanases, lipases, proteases, and pectinases can be found [28,46,68,155]. These enzymes intervene by degrading the structure and stability of fungal walls, causing their rupture and subsequent cell death [59,149]. Among the major components that shape fungal cell walls, glycoproteins and polysaccharides stand out. The latter usually represent up to 80% of the fungal cell wall, with chitin and glucan being the main ones [31]. Faced with these two components, lytic enzymes intervene by breaking the glycosidic bonds that provide rigidity to the wall, triggering its lysis and cell death [28]. The genes related to the synthesis of these lytic enzymes are diverse, as are their structures and modes of action. Among them is the gene amyN, which is involved in the production of amylases in *B. licheniformis* [172]; Banpr, associated with the synthesis of proteases in *B. amyloliquefaciens* [192]; chiA74, responsible for chitinase production in *B. thuringiensis* [193]; and Cel1-Cel10, related to cellulase synthesis in *B. paralicheniformis* [194]. There are numerous cases reporting the ability to produce lytic enzyme by species of the genus *Bacillus*, such as *B. subtilis*, *B. thuringiensis*, *B. licheniformis*, *B. safensis*, *B. pumilus*, *B. clausii*, *B. velezensis* and *B. cereus* [28] (Table 8).

##### Induces Systemic Resistance

Faced with the constant threat of pests and diseases that negatively impact agricultural production, plants have two ways to mediate their defensive response depending on the origin of the environmental stimulus. One of these pathways is the acquired response system (SAR: Systemic Acquired Resistance), whose activation is triggered by the irruption of a phytopathogen attack [195]. On the other hand, the Induced Systemic Resistance (ISR) is triggered by microorganisms in the plant rhizosphere, which are beneficial to the plant and are not pathogenic in nature [34,61,147]. Thus, ISR is defined as a physiological stage that involves the improvement of the innate defense system of the plant against the attack of bacteria, fungi, viruses, nematodes, insects and even abiotic stress situations such as drought, salinity and the presence of heavy metals, since they can share the same response mechanisms [196]. Regarding the mode of ISR induction, it is known that during PGPR-plant interaction, ethylene and jasmonic acid signaling pathways are activated [29,197]. As a consequence of the action of these phytohormones and the release of organic acids, there is an increase in the secretion of certain enzymes such as catalase, peroxidase (PO), superoxide dismutase (SOD), ascorbate peroxidase (APX), chitinase, lipoxygenase (LOX), β-1-3-gluconase (GLU), polyphenol oxidase (PPO) and phenylalanine ammonium lyase (PAL), thus generating tolerance to the biotic stress to which the plant is being subjected [198]. However, the induction of this defense system is not limited to the above actions, as volatile organic compounds such as alkanes, terpenoids, alcohols, phenolic compounds and ketones are also released, in addition to secondary metabolites such as antibiotics and siderophores [48]. Thus, the ISR response has a systemic, non-specific character on a broad spectrum of pathogens that, unlike SAR, does not elicit a hypersensitive response or visible symptoms such as necrosis in the plant specimen [197,199].

Based on the previous results, there is evidence that strains such as *B. amyloliquefaciens*, *B. subtilis*, *B. pasteurii*, *B. cereus*, *B. pumilus*, *B. mycoides*, and *B. sphaericus* have been responsible for ISR activation against plant pathogens under field and greenhouse conditions in several plant species such as tobacco, cucumber, melon, arabidopsis, and tomato, among other crops [200] (Table 8).

##### Antibiosis

Antibiosis is one of the most widespread biocontrol mechanisms by species of the genus *Bacillus* used to inhibit infections by plant pathogens [34,149]. These rhizobacteria produce several secondary metabolites that are secreted during the sporulation and stationary development phases [29]. These metabolites—termed antibiotics—are low molecular weight organic compounds, which at low concentrations (<10 ppm) are toxic to other microorganisms of pathogenic nature as they interfere with their cell wall synthesis, membrane structure, protein synthesis and inhibiting respiratory chain enzymes [32,196]. According to the data obtained in several previous investigations, its activity against Gram-positive bacteria and Gram-negative bacteria, as well as against phytopathogenic fungi such as *Aspergillus flavus*, *Fusarium oxysporum*, *Alternaria solani*, *Botrysphaeria ribis*, *Helminthosporium maydis*, *Phomopsis gossypii* and *Colletotrichum gloesporioides* is known [201]. Being a heterogeneous group of compounds, they can be classified according to their synthesis origin—into ribosomal or non-ribosomal [28,68,202]. The first group comprises bacteriocins, whereas the non-ribosomal group comprises lipopeptides, peptides and polyketides, which are synthesized by ribosome-independent enzymes that mediate the condensation of amino acid residues [35]. Regarding bacteriocins, their action is focused on interfering with cell wall synthesis, forming pores in the cell membrane and inducing cell lysis [28,203]. Some authors argue that they have a narrow spectrum of action, being directed towards species of the same or related genera [35], while other research shows that bacteriocin-producing *Bacillus* species exhibit a broad spectrum of antimicrobial activity, including bacteria, fungi, oomycetes and viruses [45,68]. Bacteriocins or derivatives thereof produced by *Bacillus* include amylosin, amycin, subtilin, subtilosin A, subtilosin B or turicin, which have been isolated from various species such as *B. amyloliquefaciens*, *B. subtilis*, *B. thuringiensis*, *B. cereus*, and *B. coagulans* [204]. Among these bacteriocins, megacin from *B. megaterium* and subtilin from *B. subtilis* are among the most studied and characterized [205]. The group of genes involved in the synthesis of the latter includes the structural gene coding for the precursor, spas, as well as other genes such as SpaB and SpaC which code for a dehydratase and a cyclase respectively—proteins involved in the posttranslational modification of the precursor. In addition, genes for both transport and secretion of the modified precursor such as SpaT and SpaFEG are included, with conversion of the modified precursor to the mature subtilin by nonspecific proteases. In addition, this group includes a gene for immunity against subtilin itself [204]. Thus, the isolation and characterization of bacteriocins can be important in the control of bacterial pathogens.

On the other hand, the so-called cyclic lipopeptides stand out for their antifungal activity. These compounds are organized as amino acid rings with fatty acid side chains that show great heterogeneity [206]. Their structural diversity allows them to act as control agents for numerous microorganisms [34]. According to this structural heterogeneity, they are divided into three families: fengicins, surfactins and iturins, which can be produced individually or jointly depending on the *Bacillus* species [28,34]. Moreover, like other secondary metabolites, the synthesis of these bioactive molecules is regulated by phosphate, stimulating their production under phosphate-limiting conditions. It has been shown that transcriptional control by phosphate is mediated by a two-component system, *PhorR* and *PhoP*, which in turn regulate the alkaline phosphatase gene, *PhoA* [207]. This is why the affection of these genes can also influence the expression of the genes for the synthesis of these lipopeptides. The synthesis of these molecules is not only directed by several groups of genes such as srf, bmy, fen, nrs, dhb, mln, bae and dif, which give rise to molecules such as surfactin, bacillomycin, fengicin, bacillibactin, macrolactin, bacillaene and difficidin [208], but also require 4′-phosphopanteteinyl transferase, a product of *sfp* gene expression which is crucial in the synthesis of non-ribosomal peptides and thus lipopeptides [209] (Table 8).

Their methods of dealing with phytopathogens are based on producing changes in the cell membrane of the pathogen, either by affecting its structure or its permeability through disruption, solubilization or pore formation [203]. In the case of phengicins, these are known to inhibit many filamentous fungi by rupturing their cell membrane and causing their death [34]. These are produced by five non-ribosomal peptide synthetases—Fen1–Fen5—encoded respectively by fenA-E genes [210]. On the other hand, surfactins contribute to swarming and biofilm formation by *Bacillus* [211]. They have a broad spectrum of action on bacteria and fungi by preventing numerous fungal diseases [212]. In the case of these lipopeptides, their non-ribosomal synthesis is due to surfactin synthetase, produced thanks to the expression of the srfA gene, which consists of four open reading frames—srfAORF1–srfA0RF4—each of them coding for each of the enzymatic modules that make up this enzyme, with molecular weights of 402, 401, 144 and 44 kDa, respectively [213]. It is worth mentioning that phengicins and surfactins have been characterized as inducers of plant ISR [29]. The synthesis of these molecules is also regulated by four open reading frames that are regulated by the Pitu promoter. These are ituA, responsible for the synthesis of β-fatty amino acids; ituB, which encodes a peptide synthetase, as does ituC; and ituD, responsible for malonyl-CoA transacylase activity, which is related to fatty acid synthesis [214]. Miljakovic et al. [28] reported the existence of *Bacillus* species that produce antibiotics of non-ribosomal origin. These include peptides (bacillisin, rhizocticin, amicoumacin, mycobalicin, and diketopiperazines) and polyketides (bacillene, dihydrobacillene, and macrolactin), which demonstrate significant antifungal and antibacterial activity. In recent years, there have been several research groups that have devoted their efforts to characterize the antibiotics produced by *Bacillus* spp., seeking to identify which pathogen they face, their mode of action, their genetic coding, and other relevant parameters of their nature (Table 8).

##### Biofilm Formation

In terms of pest and disease biocontrol, research efforts often focus on the ability of PGPRs to induce ISR, produce lytic enzymes, or produce antibiotics as mechanisms to combat plant pathogens [35]. However, the ability to form biofilm by PGPRs has also gained importance as a tool for biocontrol [170]. The control in *Bacillus* species of flagellar motility and biofilm formation is regulated by the two-component DegU-DegS system. The DegQ protein increases the phosphorylation of DegU, thus causing the increase in the aforementioned processes [215]. Biofilms are masses of colonies of one or several species of microorganisms closely packed with each other, embedded in an extracellular matrix of self-produced polymeric substances that may be attached to a surface of biotic or abiotic origin [216,217]. The bacterial biofilm has several roles such as adhesion, cohesion and aggregation of soil particles, retention of water molecules, and facilitation of ionic and genetic exchange, among others [218]. At the same time, plant health is benefited by the presence of biofilm by causing increased resistance to antibiotics, chemicals, heat, radiation, and other environmental stresses [47]. Thus, biofilm forming PGPRs possess a stable attachment and persistence mechanism in plant roots that may be advantageous to the plant by being able to inhibit other competing organisms, enhance nutrient acquisition, and adapt to environmental changes by improving tolerance to abiotic stresses [170,216]. All these reasons justify root colonization by biofilm as a preventive measure for pathogen biocontrol [30]. There are studies of numerous *Bacillus* species such as *B. velezensis*, *B. atrophaeus* and *B. subtilis* that are able to colonize roots and create biofilms as a biocontrol strategy [29,30]. Thus, the above shows the importance of biofilm formation by PGPR, its relevance in the genus *Bacillus* and its potential for colonization and competition with other organisms for interaction with the plant (Table 8).

##### Volatile Organic Compounds Production

One of the indirect mechanisms that PGPRs possess to enhance plant development and provide tolerance to various biotic and abiotic stresses is the production of volatile organic compounds (VOCs) [40]. VOCs are low molecular weight compounds (≤300 Da), with a low boiling point, which can travel long distances through soil and air due to their high vapor pressure and their ability to diffuse through air and through water-filled pores in soil at ambient temperature [29]. They originate from catabolic pathways such as glycolysis, proteolysis, fermentation, organic acid biosynthesis and sulfur metabolism [219]. Their main role is to exert a signaling function through plant-bacteria chemical interactions in the rhizosphere zone [40]. Among these signaling processes, the activation of the plant ISR stands out, thus promoting an induction in the innate plant defense system, tolerance to abiotic stresses (drought, salinity and presence of heavy metals) and reporting an improvement in plant development [171,172]. In addition to the mentioned functions of VOCs, they play an important role in the bacterial life cycle (motility, antibiotic resistance, biofilm formation) and in plant development (biomass increase, productivity, seed production, lateral root formation, nutrient acquisition and photosynthetic activity) [11,30].

Regarding the production of VOCs by *Bacillus* species, there are numerous studies describing substances such as aldehydes, alcohols, ketones, alkanes, ethers, fatty acids, phenolic compounds and jasmonates [35,40] (Table 8).

##### Quorum Sensing

Communication between individuals of bacterial populations is a key factor in the interactions that will develop between different species, whether beneficial or detrimental. The mechanism that allows the development of this intercellular relationship between bacteria is known as quorum sensing [29,170,220]. This is based on the detection of and response to signaling molecules that are secreted extracellularly by bacteria. These low molecular weight molecules are called autoinducers. These increase in concentration as the bacterial density increases in the environment, thus activating a signaling cascade that induces the expression of certain bacterial genes [149]. Among the most studied autoinducers are the N-acyl-l-homoserine lactones (AHLs: N-acyl-l-homoserine lactones) [221]. Quorum sensing controls genes related to processes including bioluminescence, sporulation, competence, antibiotic production, biofilm formation and secretions related to virulence factors [34]. In the rhizosphere, quorum sensing becomes particularly relevant as it is related to the activation of biofertilization, biocontrol and bioremediation processes [170]. This mechanism known as quorum quenching consists of the blocking of quorum sensing thanks to the degradation of the autoinductors through the use of specific enzymes such as lactonases, oxidoreductases and acylases [29,220]. The degradation by lactonases of autoinducers is based on a hydrolysis of the lactone ring of the AHL. This process can also occur spontaneously in the presence of an alkaline pH and can be reversible when the pH is acidified. Meanwhile, degradation by acylases is based on the cleavage of the amide bond and the generation of the corresponding free fatty acids and lactone ring. Finally, the degradation mechanism of oxidoreductases is based on the oxidation and thus disruption of the quorum sensing signal molecules [221]. In this way, the pathogen is rendered less virulent and negatively affects plant growth [155]. Quorum sensing and quorum quenching are vital in the development of PGPR abilities. The interaction between the rhizobacteria themselves for the activation of numerous processes that confer biofertilization, biostimulation and biocontrol capabilities is critical [222]. The PGPR of the *Bacillus* genus are positioned with special relevance in the study of the quorum sensing and quorum quenching mechanism (Table 8). Thus, the role of bacterial intercellular communication in good root colonization and the subsequent expression of genes that control the direct and indirect processes of plant growth promotion is evidenced. Above all, quorum quenching stands out as a defense mechanism against pathogens in the struggle to mitigate their effects and virulence on the affected plant species.

##### Hydrogen Cyanide Production

Among the numerous biocontrol mechanisms that PGPR can present to deal with plant pathogens is the production of hydrogen cyanide (HCN) [223]. The synthesis of this product is known as cyanogenesis, which is controlled by the HCN synthetase enzyme. This is associated with the plasma membrane of certain rhizobacteria allowing the transformation of the precursor glycine into the final product [224,225]. The *henA*, *henB* and *henC* genes are responsible for the synthesis of HCN synthetase [226]. The biocontrol activity of this volatile compound is based on the suppression of phytopathogen growth by interfering with the electron transport chain [5]. According to known data, HCN is able to inhibit the enzyme cytochrome c oxidase and other metalloenzymes of phytopathogens [68]. This disrupts the electron transport chain and thus the energy supply to the cells, ultimately leading to cell death [227]. The low levels of bacterial HCN production means that the compound acts in synergy with other biocontrol mechanisms such as antibiotics or hydrolytic enzymes to cope with biotic stress [68]. However, there are doubts regarding the characteristics of HCN as a bioprotective agent and some researchers suggest an approach more focused on acting as a metal chelating agent and increasing phosphorus availability, thus exerting a role more related to biofertilization [228]. Currently, the existence of numerous bacterial species is known, such as *Alcaligenes*, *Aeromonas*, *Bacillus*, *Pseudomonas* and *Rhizobium* [68], which produce HCN as a secondary metabolite, although mainly the genera *Pseudomonas* and *Bacillus* stand out in this facet [5,223].

The genus *Bacillus* is presented as one of the exponents in HCN production in plant growth-promoting rhizobacteria (Table 8).

## 5. Bacillus-Based Strategies for Enhancing Crop Production

The economic impact of microbial inputs can be conceptualized through two distinct metabolic investment strategies that differ fundamentally in their resource demands and cost–benefit profiles.

High-resource strategies involve microbial processes that require substantial metabolic investment from both the microorganism and host plant, typically directed toward acquiring and mobilizing growth-limiting nutrients. These interventions demand significant energy expenditure and are characterized by the direct modification of soil nutrient bioavailability through processes such as biological nitrogen fixation, phosphate solubilization, and potassium mobilization. Due to their high energetic costs, these strategies generate optimal economic returns primarily under conditions of severe soil nutrient limitation where alternative nutrient sources are unavailable or prohibitively expensive [229].

In contrast, low-resource strategies encompass microbial interventions with comparatively lower metabolic demands that enhance plant performance through bioregulatory mechanisms rather than direct nutrient provisioning. These include general plant growth-promoting rhizobacteria (PGPR) activities such as, phytohormone production, stress tolerance enhancement and biocontrol strategies that prevent pathogen-induced yield losses. Low-resource approaches are generally considered more cost-effective across diverse agroecological contexts because they require minimal metabolic investment while generating significant protective or stimulatory benefits.

Empirical evidence supports distinct economic value propositions for each strategy. High-resource nutrient acquisition strategies are most economically justified under severe soil nutrient limitation, where they mobilize the otherwise unavailable nitrogen and phosphorus essential for plant growth [229]. Conversely, low-resource PGPR applications have consistently improved yields across diverse agricultural systems, including drought-prone regions where their bioregulatory functions enhance stress tolerance without substantial metabolic costs [230,231]. Biocontrol strategies exemplify the high return-on-investment potential of low-resource approaches by preventing yield losses that would otherwise negate gains from improved nutrient availability, particularly under significant disease pressure [232]. This protective mechanism preserves yields generated through nutrient availability, underscoring the economic imperative of pathogen control as a complement to nutritional interventions.

The effectiveness and strategic value of microbial systems are strongly influenced by existing soil fertility, particularly the availability of synthetic fertilizers [233]. When fertilizer inputs are limited, and macronutrients such as nitrogen and phosphorus are scarce, plant yield responses rely primarily on resource-intensive acquisition strategies. In these scenarios, establishing efficient, high-cost symbioses is critical for overcoming growth limitations and achieving optimal productivity. Conversely, when exogenous fertilizers provide sufficient nitrogen and phosphorus, the benefits of high-cost microbial symbioses diminish [233,234]. In such cases, the advantages shift toward low-cost PGPR functions, including disease suppression, stress tolerance, and enhanced root architecture [235].

*Bacillus* species are especially valuable for enhancing crop resilience and stability, as they provide low-cost benefits even under high-input conditions [235]. The combination of durability, low resource requirements, and effective stress mitigation makes *Bacillus* PGPR crucial for maximizing marginal productivity and improving crop resilience, even when high chemical fertilizer inputs reduce the need for nutrient acquisition [236,237]. Nevertheless, numerous uncertainties remain to be addressed to ensure that their production and application yield the intended impact on agriculture. Consequently, the following section delineates the subsequent steps and strategies required for the rigorous selection of strains, their formulation and scale-up, and their performance. Furthermore, it emphasizes the paramount importance of biosafety throughout the handling chain—from the researcher to the end-user—while addressing the current regulatory constraints governing the development of this industry.

## 6. Future Research Directions

Future research should prioritize translating robust laboratory findings into consistent field performance by addressing three core areas. A significant challenge is the inconsistent efficacy of PGPR under uncontrolled field conditions. Progress will require extensive, large-scale, multi-location field trials across diverse agro-ecological zones to validate efficacy and refine application protocols, including seed, soil, and foliar methods. Additionally, research should investigate the influence of indigenous soil microbial communities and environmental stressors, such as soil pH and moisture, on the establishment and persistence of inoculated strains.

Elucidating the molecular and biochemical pathways underlying the beneficial effects of PGPR is essential. This effort must extend beyond confirming the presence of beneficial genes to include evaluation of kinetic rates and quantitative activities of key enzymes. Such an approach will facilitate the selection of the most effective commercial strains. Additionally, it is necessary to clarify the complex hormonal interactions, such as those involving indole-3-acetic acid (IAA), ethylene, and abscisic acid (ABA), as well as signal transduction pathways, such as induced systemic resistance (ISR) activation, which occur within the host plant following microbial exposure.

Metabolomics and advanced analytical techniques should be used to characterize the chemical diversity of root exudates and to determine how PGPR strains modify them to recruit beneficial microorganisms or suppress pathogens, thereby enabling rhizosphere engineering. Since most individual strains lack all necessary plant growth-promoting traits, rhizosphere engineering represents a logical next step. Future research should focus on designing and evaluating synthetic microbial communities with complementary and synergistic functions, such as combining nitrogen-fixers with phosphorus-solubilizers, to maximize field effectiveness.

The development of superior *Bacillus*-based bioproducts requires systematic, multi-criteria strain selection approaches that extend beyond single-trait screening. Future selection strategies must integrate quantitative performance metrics, multifunctional trait profiling, environmental fitness assessment, and biosafety considerations from the outset. Rather than merely confirming the presence of beneficial genes such as *nifH*, *phoD*, or *acdS*, selection protocols should evaluate kinetic rates and quantitative activities of key enzymes under conditions that mimic target agricultural environments, as this will facilitate the identification of the most effective commercial strains. High-throughput screening platforms combining genomic profiling with phenotypic assays will accelerate the identification of elite strains capable of exhibiting synergistic combinations of mechanisms, such as phosphorus solubilization coupled with induced systemic resistance activation and drought tolerance enhancement. Additionally, competitive rhizosphere colonization capacity, stress tolerance (temperature, pH, salinity), and compatibility with indigenous microbial communities must be evaluated during the selection process, with particular attention to endospore resilience and germination efficiency under field conditions—traits often overlooked in laboratory studies. The integration of omics-based approaches with machine learning algorithms will enable prediction of strain performance across diverse environmental conditions and identification of optimal trait combinations. Given the presence of opportunistic pathogens within the *Bacillus* genus, rigorous biosafety screening must be incorporated into selection workflows, including assessments of pathogenic potential, antibiotic resistance gene profiles, and toxin production capabilities.

Translating promising *Bacillus* strains into commercially viable bioproducts requires overcoming significant formulation and delivery challenges that remain underexplored in current research. While *Bacillus* endospores offer inherent advantages in environmental stability compared to the vegetative cells of other PGPR genera, formulation design critically influences product efficacy and commercial viability. Carrier matrix selection, including peat, talc, biochar, or polymeric encapsulation systems, directly affects spore viability and release kinetics in the rhizosphere. The incorporation of osmoprotectants, germination stimulants, or adjuvants may enhance field establishment, though systematic evaluation of these formulation components across diverse environmental conditions remains limited. Maintaining high viable spore counts (>10^8^ CFU/g) throughout product storage and distribution presents ongoing challenges, necessitating research into factors affecting long-term stability, including moisture content, storage temperature, packaging materials, and potential synergistic or antagonistic interactions when co-formulating multiple *Bacillus* strains or combining with chemical inputs.

Application method optimization represents another critical area requiring investigation. The efficacy of *Bacillus* inoculants varies significantly with application timing and delivery method, yet comparative studies evaluating seed treatment, in-furrow application, foliar sprays, and drip irrigation delivery across different crops and growth stages remain scarce. Understanding spore germination triggers and rhizosphere colonization dynamics will inform optimal application timing relative to plant phenology. Furthermore, ensuring compatibility with conventional fertilizers, pesticides, and other biostimulants commonly used in integrated crop management is essential for commercial success, as potential antagonistic or synergistic interactions may significantly impact field performance and farmer adoption.

The successful adoption of *Bacillus*-based bioproducts requires their seamless integration into existing cropping systems, presenting both technical and socioeconomic challenges. These inoculants must function effectively within the context of current farming practices, including synthetic fertilizer regimes, chemical pest management, and tillage operations. Critical research gaps include determining optimal fertilizer reduction rates when using *Bacillus* biofertilizers to maximize economic benefits without compromising yields, evaluating compatibility with fungicides and insecticides commonly applied in target crops, and assessing impacts of soil disturbance on inoculant persistence and re-colonization dynamics.

The future of PGPR application lies in precision agriculture approaches that integrate soil microbial diagnostics, environmental sensors, and predictive models to enable site-specific inoculant selection and application. Artificial intelligence-driven platforms capable of recommending optimal *Bacillus* strains and application rates based on real-time soil conditions, weather forecasts, and crop nutritional status represent a logical extension of current precision agriculture technologies. Such tools would facilitate the transition from blanket application strategies to targeted interventions tailored to specific field conditions.

While *Bacillus* bioproducts align well with organic certification requirements and regenerative agriculture principles emphasizing soil health, their efficacy in low-input systems requires further validation. Understanding how *Bacillus* inoculants interact with cover crops, compost amendments, and reduced tillage practices will facilitate their adoption in sustainable farming systems. Importantly, even superior bioproducts will fail without addressing farmer adoption barriers through techno-economic analyses demonstrating return on investment across diverse scenarios and extension programs providing practical guidance on storage, handling, and application tailored to both smallholder and large-scale operations.

If genetic engineering tools, particularly CRISPR-Cas9, are adopted to modify wild-type or near-commercial strains for enhanced traits, these modifications are expected to produce strains with greater competitive fitness and sustained functionality. However, such advancements will introduce complex regulatory challenges as authorities work to classify and register strains exhibiting multiple functions. Globally harmonized regulatory pathways—including a functional definition of plant biostimulants—are necessary to facilitate the registration of innovative, multifunctional microbial products and to address public acceptance issues related to genetically modified organisms (GMOs). As mentioned in the introduction, European Regulation 2019/1009 contains a definition for plant biostimulants. The benefits of this regulation include the introduction of harmonized standards whereby all products bearing the CE mark meet the same strict requirements in terms of quality, safety and labeling throughout the EU. In addition, biostimulants are now officially recognized and regulated within a harmonized framework. The regulation encourages the use of recycled and bio-based raw materials. Finally, it provides clearer and more standardized information on product function and claims for farmers. However, there are limitations when it comes to using microbial plant biostimulants. The legal basis of this regulation limits the use of microorganisms to only four genera permitted in CMC 7 (Component Material Category 7): *Azotobacter* spp., *Rhizobium* spp., *Azospirillum* spp. and mycorrhizal fungi. Many microorganisms are excluded, such as *Bacillus* or *Pseudomonas*. If a product developed in Europe does not exclusively include the permitted genera, its marketing within the territory is restricted. This biostimulant must comply with the legal framework of each Member State in which it is to be distributed, and its access to the single European market is restricted pending an extension of the CMC7 list. The European Union must resolve this issue through future amendments to the regulation, as the benefits of this legal framework, which ensures standardization in production, quality and product safety, do not seem to be in line with the biostimulant manufacturing industry.

Biosafety is the set of procedures required to prevent biological losses to humans and the environment. EU 2019/1009 assesses the safety of microbial strains by certifying the absence of pathogenicity. To this end, strains must not possess virulence genes or produce toxins. In addition, the absence of transferable antibiotic resistance and the control of microbial contaminants during production are required, with validated detection methods and controls at each production stage. The biosafety of a biostimulant begins in the first phase of the process. The selection of strains involves their identification. Classic rRNA identification is not sufficient. Correct phenotypic, chemotaxonomic, and genomic analyses are useful for accurately assessing the risk of each strain. The classification of microorganisms according to their risk group, as established by the World Health Organization, allows only BSL-1 (Biosafety Level-1) microorganisms to be approved for commercial use. It is also pertinent to screen for ARGs (Antibiotic Resistance Genes) and MGEs (Mobile Genetic Elements) before approving their agricultural use, to confirm that they are free of virulence genes. The Environmental and Human Safety Index (EHSI) is another resource to consider. It allows for a comparison of isolated strains with the standard scale of PGPRs recognized as having pathogenic or harmful effects on all participants in the ecosystem. Although the *Bacillus* genus has high potential, it includes species that are facultative or opportunistic human pathogens; some *B. cereus* strains have been classified as BSL-2 organisms associated with human disease. Therefore, a thorough polyphasic approach of microbial taxonomy of all strains intended for commercial use is required to reliably distinguish non-pathogenic, beneficial strains from potential human health and environmental threats.

## Figures and Tables

**Table 1:** Some examples of commercial products based on *Bacillus* sp. as biocontrol agents [41].

*Bacillus* Strain.	Commercial Product	Use
*B. amyloliquefaciens* MBI	Serifel^®^	Fungicide
*B. amyloliquefaciens* FZB24	Taegro^®^	Fungicide
*B. amyloliquefaciens* subsp. *plantarum* D747	AMYLO-X^®^ WG	Fungicide
*B. amyloliquefaciens* subsp. *plantarum* D747	VALCURE^®^	Fungicide
*B. firmus* I-1582	Flocter^®^	Nematicide
*B. pumilus* QST 2808	Sonata^®^	Fungicide
*B. subtilis* QST 713	Serenade^®^	Fungicide
*B. thuringiensis* subsp. *aizawai*	TUREX^®^	Pesticide
*B. thuringiensis* subsp. *israelensis* AM65-52	Gnatrol^®^	Pesticide
*B. thuringiensis* subsp. *kurstaki* EG 2348	Lepinox^®^	Pesticide

**Table 2:** Summary of phytohormone mechanisms, target crops, representative bacillus strains, and key effects.

Phytohormone	*Bacillus* sp.	Target Crop	Key Effect	Reference
IAA	*B. licheniformis* *B. subtilis*	Tomato (*Solanum lycopersicum*)	Increase seed germination and plant growth	[73]
IAA	*B. megaterium*	Rice (*Oryza sativa*)	Increase plant growth	[74]
IAA	*B. velezensis* *B. subtilis* *B. amyloliquefaciens*	Pepper (*Capsicum chinense*)	Increase plant growth and chlorophyll content	[75]
IAA	*B. cereus*	Wheat (*Triticum aestivum*)	Increase plant growth, grain, and crop productivity	[76]
IAA	*B. thuringiensis*	Tomato, Cotton and sugarcane (*Solanum lycopersicum*, *Gossypium hirsutum* and *Saccharum officinarum)*	Increase plant growth	[77,78,79,80]
IAA	*B. mycoides*	Cherry (*Prunus cerasus* L.)	Increase plant growth	[81]
N_2_ Fixation, siderophores and IAA	*B. siamensis*	Tomato (*Solanum lycopersicum*)	Increase plant growth under saline conditions	[26,82]
Zeatin, zeatin riboside, isopentaladenine and isopentaladenine	*B. subtilis*	Lettuce (*Lactuca sativa*)	Increase plant growth	[83]
Zeatin, cis-zeatin and isopentaladine	*B. toyonensis*	Tomato (*Solanum lycopersicum*)	Increase plant growth	[84]
Zeatin ribose y Zeatin	*B. lichenoformis* *B. subtilis*	Cucumber (*Cucumis sativus*)	Increase weight and size of cotyledons	[85]
Cytokinin and IAA	*B. amyloliquefaciens*	Arabidopsis (*Arabidopsis thaliana*)	Increase lateral root and hair-root formation	[86]
GA_1_, GA_3_, GA_5_, GA_8_, GA_19_, GA_23_ and GA_24_	*B.tequilensis*	Soy (*Glycine max*)	Tolerance to heat stress	[87]
GA_1_, GA_3_, GA_7_, GA_8_ and GA_20_	*B. methylotrophicus*	Strawberry (*Fragaria x ananasa*) Lettuce (*Lactuca sativa*)	Increase plant growth	[88]
ABA	*B. amyloliquefaciens*	Rice (*Oryza sativa*)	Tolerance to saline stress	[89]
ABA and IAA	*B. amyloliquefaciens*	Oil palm (*Elaeis guineensis*)	Increase nutrient uptake and AIA and ABA content	[90]
ABA	*B. mirasflavi*	Mustard (*Brassica juncea*)	Tolerance to drought stress	[91]
ABA	*B. subtilis*	Chinese cabbage (*Brassica chinensis*)	Tolerance to Cd stress	[92]
ACCd	*B. mojavensis*	Wheat (*Triticum aestivum*)	Tolerance to saline stress	[93]
ACCd	*B. subtilis* *B. safensis*	Wheat (*Triticum aestivum*)	Tolerance to saline stress	[94]
ACCd	*B. subtilis*	Barley (*Hordeum vulgare*)	Tolerance to saline stress	[95]
ACCd	*B. aryabhattai*	Mustard (*Brassica juncea*)	Tolerance to heat stress	[96]

**Table 3:** Summary of siderophore mechanisms, representative *Bacillus* strains, target crops, and key effects.

*Bacillus* sp.	Target Crop	Key Effect	Reference
*B. siamensis*	Chickpea (*Cicer arietinum*)	Increase plant growth	[26]
*Bacillus* spp.	Bell Pepper and Maize (*Capsicum* spp. and *Zea mays*)	Increase seed germination	[55]
*B. mycoides*	Maize (*Zea mays*)	Increase plant growth	[110]
*B. cereus*	Tomato (*Lycopersicon esculentum*)	Increase plant growth	[111]

**Table 4:** Summary of salt stress research, representative *Bacillus* strains, target crops, and key effects.

*Bacillus* sp.	Target Crop	Key Effect	Reference
*B. paralicheniformis* TRQ65	Wheat (*Triticum aestivum*)	Promoted growth under saline conditions.	[129]
*B. frigotolerans* (alone or co-inoculated)	Wheat (*Triticum aestivum*)	Alleviated salt stress and improved wheat development.	[130]
*B. licheniformis* A2	Groundnut (*Arachis hypogea*)	Stimulated growth in the presence of salt stress.	[131]
*B. aryabhattai* PM34	Wheat (*Triticum aestivum*)	Improved growth and tolerance to salt stress in laboratory experiments.	[132]
*B. halotolerans* KKD1	Wheat (*Triticum aestivum*)	Modulated plant responses to salt stress.	[120]
*B. megaterium* OQ560352	Maize (*Zea mays*)	Stimulated plant growth and induced resistance under saline soil conditions.	[133]
*B. amyloliquefaciens* E50S2-3 and *B. velezensis* M100S1-4	Rice (*Oryza sativa*)	Improved plant parameters under conditions of salt stress and pollutant residues.	[134]

**Table 5:** Summary of temperature stress research, representative bacillus strains, target crops, and key effects.

Temperature Stress	*Bacillus* sp.	Target Crop	Key Effect	Reference
Heat	*B. tequilensis* SSB07	Soybean (*Glycine max*)	Greatly enhanced biomass, size, leaf development, and photosynthetic pigment content when exposed to high temperatures.	[87]
Heat	*B. cereus*	Tomato (*Solanum lycopersicum*)	Mitigated the adverse effects of heat by promoting exopolysaccharide production and reducing ACC content.	[138]
Heat	*B. subtilis*	Beans (*Phaseolus vulgaris* L.)	Mitigated the adverse effects of high temperatures (35 °C) by promoting growth and development during vegetative and reproductive stages.	[139]
Heat	*B. licheniformis* BE-L60	Spinach (*Spinacia oleracea* L.)	Led to better plant balance and triggered the antioxidant system, improving plant health under heat stress.	[140]
Cold	Several *Bacillus* strains	Wheat (*Triticum aestivum*)	Demonstrated improved responses to cold stress by regulating abscisic acid, lipid peroxidation, and proline accumulation pathways.	[141]
Cold	*B. amyloliquefaciens* GL18	Oat seeds (*Avena sativa*)	Increased biometric parameters and levels of phytohormones (salicylic acid, jasmonic acid, abscisic acid), confirming tolerance to low temperatures (4 °C).	[142]
Cold	*B. methylotrophicus* VL-10	Tomato (*Solanum lycopersicum*)	Promoted growth, improved the defensive response, and reduced root shock response under cold conditions (15 °C/8 °C).	[143]

**Table 6:** Summary of drought stress research, representative bacillus strains, target crops, and key effects.

*Bacillus* sp.	Target Crop	Key Effect	Reference
*B. subtilis* strain GOT9	*Arabidopsis thaliana* and *Brassica campestris*	Improved lateral root development in Arabidopsis and regulated genes involved with osmotic stress.	[150]
*B. licheniformis* and *B. megaterium* strains	Wheat (*Triticum aestivum*)	Increased germination index (11–46%), seed vigor index (11–151%), fresh weight (35–191%), and increased relative content of water, photosynthetic pigments, and osmolytes.	[151]
*B. amyloliquefaciens* MMR04	Millet (*Pennisetum glaucum*)	Improved growth parameters, chlorophyll content, and relative water content under drought stress; use of an antioxidant system was observed.	[152]
*B. amyloliquefaciens* strain QST713	Alfalfa (*Medicago sativa* L.) (tolerant and sensitive varieties)	Improved plant development compared to controls; modified relative water content, chlorophyll accumulation, and antioxidant enzyme activities.	[153]
*B. amyloliquefaciens* E50S2-3 and *B. velezensis* M100S1-4	Rice (Oryza sativa)	Improved plant parameters under conditions of drought stress and pollutant residues.	[134]

**Table 7:** Summary of heavy metals stress research, representative bacillus strains, target crops, and key effects.

Heavy Metal	*Bacillus* sp.	Target Crop	Key Effect	Reference
Cr and Cd	*B. anthracis* PM21	Egyptian riverhemp (*Sesbania sesban*)	Withstood metal stress (Cr and Cd) by maintaining homeostasis through antioxidant activities, resulting in increased growth and biomass.	[164]
Cr	*B. subtilis* strain	Wheat (*Triticum aestivum*)	Combined with phosphorus fertilizer, reduced the accumulation of contaminants in shoots (54.24%), roots (59.19%), and grains (90.26%).	[165]
Cr	*B. cereus* strain	Black mustard (*Brassica nigra*)	Improved plant germination and development; increased incorporation, bioaccumulation, and translocation of Cr throughout the plant.	[166]
Cd	*B. cereus* and *B. megaterium*	Mustard (*Brassica juncea*)	Increased shoot and root fresh/dry weight and shoot K content; *B. cereus* minimized Cd+2 translocation/bioaccumulation, and *B. megaterium* reduced Na+ and Cd+2 in the shoot.	[167]

**Table 8:** Summary of biotic control mechanisms, representative bacillus strains, target crops, and key effects.

Mechanism	*Bacillus* sp.	Target Crop	Key Effect	Reference
Lytic Enzymes	*B. thuringiensis*	Barrel medic (*Medicago truncatula*) (*against Botrytis cinerea*)	Produces chitinases that target the pathogen.	[29]
	*B. tequilensis* PKDN31 and *B. licheniformis* PKDL10	Tomato (*Solanum lucycopersicum*) (suppressing *Fusarium oxysporum* F. sp. *lycopersici*)	Produce amylase, protease, lipase, and beta-1,3-glucanase, which suppress the pathogen.	[173]
	*B. subtilis* EG21	Potato (*Solanum tuberosum*) (against *Phytophthora infestans* and *Rhizoctonia solani*)	Synthesizes pectinases, cellulases, and chitinases in response to pathogens.	[174]
	*B. cereus* BW8	Apples and tropical fruits (against fungi)	Produces amylase and cellulase, contributing to fungal biocontrol.	[175]
Induced Systemic Resistance (ISR)	*B. thuringiensis* serovar *aizawai* ABTS-1857	Tomato (*Solanum lycopersicum* cv. Momotaro)	Controlled *Botrytis cinerea* by activating ISR and inducing defense-related gene expression.	[176]
	*B. amyloliquefaciens* Ba13	Tomato (*Lycopersicon esculentum* Mill. cv. Guofen 1)	Enhanced resistance to yellow leaf curl virus by activating ISR, improving biocontrol against the whitefly (*Bemisia tabaci*).	[177]
	*B. subtilis* SL18r	Tomato (*Solanum lycopersicum)*	Increased resistance to *Botrytis cinerea* through ISR activation.	[178]
	*Bacillus* sp. Bsp.3/aM	Chili pepper (*Capsicum annuum* L.)	Reduced anthracnose incidence by regulating defense-related enzymes (e.g., PAL, POX, PPO, LOX, chitinase).	[179]
	*B. proteolyticus* OSUB18	*Arabidopsis thaliana* (against *P. syringae* and *B. cinerea*)	Induced ISR by increasing reactive oxygen species, phytohormones, and secondary metabolites involved in defense.	[180]
	Two *Bacillus* strains	Saffron (*Crocus sativus* L.) (against *Fusarium oxysporum* R1)	Reduced disease incidence through ISR activation and high production of defense-related enzymes.	[181]
Antibiosis (Lipopeptides)	*B. velezensis* strain	Banana rhizosphere (inhibiting *Ralstonia solanacearum* and *Fusarium oxysporum*)	Produced surfactins, iturins, and fengicins, which inhibited the pathogens.	[182]
Biofilm Formation	*B. amyloliquefaciens*	Banana (*Musa AAA Cavendish* cv. Brazil)	Root exudates induced chemotaxis and biofilm formation, facilitating root colonization.	[27]
	*B. vallismortis* TR01K	Tea (*Camelia sinensis*)	Produced high levels of biofilms; associated with nutrient mobilization and plant growth-promoting traits.	[158]
Volatile Organic Compounds (VOCs)	*B. velezensis* (Produces 2,3-butanediol and acetoin)	Tobacco (*Nicotiana tabacum* cv.)	Activates ISR and causes stomatal closure in response to O3, initiating a defensive response.	[183]
	*Bacillus* sp. JC03 strain	Arabidopsis and Tomato plants	Showed significant increases in biomass and enhanced overall growth.	[184]
	*B. mojavensis* I4	Arabidopsis	VOCs demonstrated in vitro antifungal activity (*F. verticillioides*, *F. graminearum*, *R. solani*); increased chlorophyll content and biomass.	[185]
	*B. velezensis* HNA3	Various pathogens (*Alternaria alternata*, *F. oxysporum*, etc.)	Demonstrated plant growth promotion and biocontrol; VOCs (e.g., 9-octadecenoic acid methyl ester (z)) inhibited fungal growth.	[186]
Quorum Quenching (QQ)	*Bacillus* sp. isolates As30, Gs42, and Gs52	Green soybean (*Vigna radiata*)	Attenuated symptoms of *Pectobacterium carotovorum* subsp. *carotovorum* by producing AHL-degrading enzymes.	[187]
	*B. subtilis* UD1022	*Barrel medic* (*Medicago truncatul*) (interaction with *S. meliloti*)	YtnP lactonase delayed or inhibited nodulation by exerting a QQ effect.	[188]
HCN Production	*B. licheniformis* TRS-1/*B. pumilus* TRS-5	Tomato rhizosphere (against *Curvularia* sp. and *Xanthomonas* strains)	Showed antimicrobial activity against fungal and bacterial pathogens.	[189]
	*B. velezensis* Vb1, *B. paramycoides* Vb3, and *B. paramycoides* Vb6 (Consortium)	Broad bean (*Vicia faba*) (against *Fusarium oxysporum*)	Consortium improved plant resistance to the pathogen under greenhouse conditions.	[190]
	*B. megaterium* CtST3.5	Tomato (against *A. tumefaciens* and *M. incognita*)	Inhibited *Agrobacterium tumefaciens* and reduced viability of *Meloidogyne incognita* juveniles in vitro; improved tomato growth.	[191]

## Data Availability

No new data was created or analyzed in this study.

## Supplementary Materials

The following supporting information can be downloaded at: https://www.mdpi.com/article/10.3390/plants15030516/s1 [112,113,114,115,116,117,118].
