# Supplementary material for: Bacillus as Premier Biocontrol Agents: Mechanistic Insights, Strategic Application, and Future Regulatory Landscapes in Sustainable Agriculture
Source: Plants (Basel). 2026 Feb 6;15(3):516. doi: 10.3390/plants15030516 (PMC12899473; doi:10.3390/plants15030516)
Supplement: Supplementary file 1 [file plants-15-00516-s001.zip › plants-4104712-supplementary.pdf]

Table S1. Molecular Mechanisms of *Bacillus* PGPR in Plant Abiotic Stress Responses.

| Abiotic Stress Type | ***Bacillus* sp.** | **Target Crop** | **Molecular Mechanism Triggered** | **Enzymatic Activity (Inferred)** | **Impact on Plant Physiology** | **Reference** |
|---|---|---|---|---|---|---|
| Salinity | *Bacillus subtilis* | *Triticum aestivum* (Wheat) | Induced Systemic Resistance (ISR), production of compatible solutes (osmolytes) | SOD, CAT, POD | Improved plant biomass, relative water content, and reduced electrolyte leakage | 2 |
| Salinity | *Bacillus amyloliquefaciens* | *Oryza sativa* (Rice) | Regulation of salt-stressed gene expression | SOD, CAT, APX | Enhanced salinity tolerance and plant growth promotion | 2 |
| Salinity | *Bacillus amyloliquefaciens* | *Zea mays* (Maize) | Secretion of spermidine to reduce oxidative damage and Na+ toxicity | SOD, CAT, POD | Improved salt sensitivity, higher total chlorophyll accumulation, and reduced oxidative stress | 1 |
| Salinity | *Bacillus safensis PM22* | *Zea mays* (Maize) | ACC deaminase activity, EPS and siderophore production | APX, POD, SOD, CAT | Enhanced photosynthetic pigments, leaf relative water content, and salt tolerance index | 2 |
| Salinity | *Bacillus licheniformis* | *Arachis hypogaea* (Peanut) | Reduction of stress-induced ethylene via ACC deaminase | ACC deaminase | Improved growth and salt tolerance through mediation of ABA levels | 2 |
| Salinity | *Bacillus halotolerans* | *Triticum aestivum* (wheat) | Metabolic and molecular reprogramming; modulation of stress-responsive gene expression | SOD, CAT, APX | Enhanced growth under salt stress | 1,6 |
| Salinity | *Bacillus subtilis NA2* | *Triticum aestivum* (Wheat) | Modulation of antioxidants and pigments | SOD or CAT | Improved growth; enhanced levels of ascorbic acid and chlorophyll | 7 |

| | | | | | | |
|---|---|---|---|---|---|---|
| Salinity | *Bacillus amyloliquefaciens SN13* | *Oryza sativa* (Rice) | Upregulation of genes for apical meristem and ABA-responsive genes | - | Promoted plant growth; adjusted carbohydrate metabolism | 7 |
| Salinity | *B. licheniformis K11* | *Arachis hypogaea* (Peanut) | - | SOD, CAT, APX | SOD, CAT, APX | 5 |
| Heat | *Bacillus cereus* | *Solanum lycopersicum* (Tomato) | Cleavage of ACC into alpha -ketobutyrate and ammonia; induction of heat shock proteins and antioxidant enzyme activities; EPS production | ACC deaminase, SOD, APX, CAT | Promoted shoot and root length; increased leaf surface area, biomass, carotenoid, and protein content | 2,3,5 |
| Heat | *Bacillus safensis* | *Triticum aestivum* (Wheat) | Enhancing activity of antioxidant enzymes and HSPs expression | SOD, CAT, or POD | Alleviated heat stress; increased protein content | 7 |
| Heat | *B. thuringiensis, B. subtilis* | *Triticum aestivum* (Wheat) | - | SOD, CAT, APX | Growth promotion under heat stress | 5 |
| Drought | *Bacillus subtilis* | *Solanum lycopersicum* (Tomato) | Production of ACC deaminase to lower ethylene levels; induction of systemic resistance | ACC deaminase, APX, SOD, CAT | Enhanced Relative Water Content (RWC), increased water use efficiency, and improved biomass and growth | 1 |
| Drought | *Bacillus subtilis* | *Triticum aestivum* (Wheat) | Accumulation of osmolytes and ACC deaminase activity | ACC deaminase | Enhanced seed germination and overall plant growth | 3 |
| Drought | *Bacillus licheniformis* K11 | *Capsicum annuum* (Pepper) | Upregulation of stress proteins (Cadhn, VA, sHSP, and CaPR-10) and ACC deaminase production | ACC deaminase | Mitigated water scarcity stress; increased root/shoot length, biomass, and survival rate | 4 |
| Drought | *Bacillus amyloliquefaciens* | *Triticum aestivum* (Wheat) | Enhanced IAA production and upregulation of stress-related genes (APX1, SAMS1, HSP17.8) | APX (Ascorbate peroxidase) | Improved yield under water stress | 4 |
| Drought | *Bacillus pumilus* | *Zea mays* (Maize) | Regulation of water absorption and root modification; production of ABA in culture media | SOD, CAT, POD | Improved tolerance to water scarcity, nutrient limitations, and chlorophyll content | 3,6 |

| | | | | | | |
|---|---|---|---|---|---|---|
| Drought | *Bacillus thuringiensis* | *Lavandula dentata* | Synthesis of Indole-3-acetic acid (IAA) | SOD, CAT, or POD | Augmented nutritional, physiological, and metabolic functions | 4 |
| Drought | *Bacillus thuringiensis* AZP2 | *Triticum aestivum* (Wheat) | Production of ACC deaminase and phosphate solubilization | ACC deaminase | Enhanced photosynthesis and biomass production | 4 |
| Drought | *Bacillus megaterium* | *Cicer arietinum* (Chickpea) | ACC deaminase synthesis and phosphate solubilization | ACC deaminase, Phosphatase | Alleviation of water deficit and improvement of biomass | 2 |
| Drought | *Bacillus mirasflavi* | *Brassica juncea* | Production of ABA analog (Xanthoxin) | SOD, POD, CAT | Modulated physiological response to water stress | 1 |
| Drought | *Bacillus licheniformis* | Vitis vinifera (Grapevine) | Modulation of plant ABA content to regulate stomatal closure | - | Reduced rate of plant water loss and enhanced stress relief | 1 |
| Drought | *Bacillus circulans ML2 and B. megaterium ML3* | Cucurbita pepo (Squash) | Osmoprotective agents | SOD, CAT, APX | Improved squash growth under stress | 5 |
| Heavy Metal (As, Cd) | *Bacillus licheniformis* | *Spinacia oleracea* (Spinach) | Enhanced expression of genes encoding antioxidant enzymes | SOD, POD, CAT | Reduced oxidative stress; improved growth | 7 |
| Heavy Metal (Pb) | *Bacillus subtilis FBL-10* | *Solanum melongena* (Eggplant) | Activation of plant antioxidant system | SOD, POD, APX | Decreased levels of MDA and peroxide | 2 |
| Heavy Metal (Cd) | *Bacillus siamensis* | *Triticum aestivum* (Wheat) | Reduced Cadmium accumulation | SOD, CAT | Improved growth and antioxidant defense | 5 |
| Heavy Metal (Cr) | *Bacillus cereus* | *Brassica nigra* | Phytoextractability improvement | SOD, CAT, APX | Improved growth in contaminated soil | 5 |
| Heavy Metal (Cd, Pb) | *Bacillus spp.* | *Solanum nigrum* | Promotion of phytoextraction | SOD, CAT, APX | Promotion of growth and phytoextraction | 5 |

| | | | | | | |
|---|---|---|---|---|---|---|
| Heavy Metal (Pb) | *Bacillus amyloliquefaciens* | *Mentha piperita* | ACC deaminase production | ACC deaminase, SOD, CAT | Minimized lead stress | 5 |

## References


1. Al-Turki, A.; Murali, M.; Omar, A.F.; Rehan, M.; Sayyed, R.Z. Recent advances in PGPR mediated resilience toward interactive effects of drought and salt stress in plants. Front. Microbiol. 2023, 14. https://doi.org/10.3389/fmicb.2023.1214845
2. Jha, Y.; Macwan, A. A.; Ghanaim, A. M.; Mohamed, H. I. Management of abiotic and biotic stresses by microbiome-based engineering of the rhizosphere. Biocatal Agric Biotechnol, 2024, 61, 103365. https://doi.org/10.1016/j.bcab.2024.103365
3. Fanai, A.; Bohia, B.; Lalremruati, F.; Lalhriatpuii, N.; Lalrokimi, Lalmuanpuii, R.; Singh, P. K.; Zothanpuia. Plant growth promoting bacteria (PGPB)-induced plant adaptations to stresses: an updated review. PeerJ, 2024, 12, e17882. https://doi.org/10.7717/peerj.17882
4. El-Saadony, M. T.; Saad, A. M.; Mohammed, D. M.; Fahmy, M. A.; Elesawi, I. E.; Ahmed, A. E.; Algopishi, U. B.; Elrys, A. S.; Desoky, E. M.; Mosa, W. F.; El-Mageed, T. A. A.; Alhashmi, F. I.; Mathew, B. T.; AbuQamar, S. F.; El-Tarabily, K. A. Drought-tolerant plant growth-promoting rhizobacteria alleviate drought stress and enhance soil health for sustainable agriculture: A comprehensive review. *Plant Stress*, **2024**, *14*, 100632. https://doi.org/10.1016/j.stress.2024.100632
5. Khan, N.; Ali, S.; Shahid, M.A.; Mustafa, A.; Sayyed, R.Z.; Curá, J.A. Insights into the Interactions among Roots, Rhizosphere, and Rhizobacteria for Improving Plant Growth and Tolerance to Abiotic Stresses: A Review. *Cells* **2021**, *10*, 1551. https:// doi.org/10.3390/cells1006155
6. Maciel-Rodríguez, M.; Moreno-Valencia, F.D.; Plascencia-Espinosa, M. The Role of Plant Growth-Promoting Bacteria in Soil Restoration: A Strategy to Promote Agricultural Sustainability. *Microorganisms* **2025**, *13*, 1799. https://doi.org/10.3390/ microorganisms13081799
7. Kumar, D.; Ali, M.; Sharma, N.; Sharma, R.; Manhas, R. K.; Ohri, P. Unboxing PGPR-mediated management of abiotic stress and environmental cleanup: what lies inside? *Environmental Science And Pollution Research*, **2024**, *31*(35), 47423-47460. https://doi.org/10.1007/s11356-024-34157-1
